# Effect of Supplementing Different Levels of L-Glutamine on Holstein Calves during Weaning

**DOI:** 10.3390/antiox11030542

**Published:** 2022-03-12

**Authors:** Shuo Wang, Fuwei Wang, Fanlin Kong, Zhijun Cao, Wei Wang, Hongjian Yang, Yajing Wang, Yanliang Bi, Shengli Li

**Affiliations:** 1 State Key Laboratory of Animal Nutrition, Beijing Engineering Technology Research Center of Raw Milk Quality and Safety Control, College of Animal Science and Technology, China Agricultural University, Beijing 100193, China; b20213040351@cau.edu.cn (S.W.); fanlinkong@cau.edu.cn (F.K.); caozhijun@cau.edu.cn (Z.C.); wei.wang@cau.edu.cn (W.W.); yang_hongjian@cau.edu.cn (H.Y.); yajingwang@cau.edu.cn (Y.W.); 2 Beijing Sunlon Livestock Development Co., Ltd., Beijing 100076, China; wang_fuwei@foxmail.com; 3 Key Laboratory of Feed Biotechnology of the Ministry of Agriculture and Rural Affairs, Institute of Feed Research, Chinese Academy of Agricultural Sciences, Beijing 100081, China

**Keywords:** weaning stress, L-Gln, calves, rumen-protected

## Abstract

Weaning stress affects the health and performance of calves. L-glutamine (L-Gln) is commonly used as a functional antioxidant and energy supplement in the body. However, dietary L-Gln supplementation improving weaning stress of calves is unclear. Thus, we aimed to explore the effects of L-Gln (provided by rumen-protected L-Gln) on calves during weaning. Seventy-five Holstein calves (54.0 ± 2.68 kg; 42 ± 2.1 d of age) were assigned to five groups: no supplementation and L-Gln with 1%, 2%, 3%, and 4% dry matter daily intake (DMI) supplementation groups, respectively. The experiment lasted for 28 days (42–70 d of age of calves), and the calves were weaned at 15 d of experiment. DMI and body weekly weight of all calves were recorded. Blood samples of nine healthy calves with similar body weight were collected from each group at 0, 7, 14, 16, 18, 21, and 28 d of experiment for detecting serum L-Gln, glucose, insulin, urea nitrogen, D-lactate, cortisol, haptoglobin, interleukin-8, immunoglobulin (Ig) G, IgA, IgM, total antioxidant capacity, superoxide dismutase, glutathione peroxidase, catalase, and malondialdehyde. At the end of the experiment, six healthy calves with similar body weight from each group were selected for slaughter and morphological analysis of small intestine tissue. The results showed that the L-Gln supplementation in the diets improved the negative effects of sudden weaning in calves. Furthermore, compared to the higher-level L-Gln supple-mentation (3 and 4% of DMI) groups, the dietary lower-level L-Gln supplementation (1 and 2% of DMI) had higher average daily gain, glutathione peroxidase and IgG concentration, and villus height/crypt depth of the duodenum and jejunum, as well as lower cortisol, haptoglobin, and interleukin-8 concentration of weaned calves. These results provided effective reference for relieving the negative effects of calves during weaning.

## 1. Introduction

In large-scale dairy farms, calves weaning as early as possible is necessary without negative effects. Not only does it increase the amount of milk available for human consumption, but it also improves dairy management and increases cow reproductive efficiency [1,2]. However, early weaning can result in significant physical and psychological damage to calves, producing weaning stress [3]. It is well known that weaning stress can reduce feed intake and damage the small intestinal structure of calves, which affects their productive capacity in adulthood [4,5,6]. 

Weaning stress is associated with significant changes in amino acid metabolism [7]. In particular, L-glutamine (L-Gln) metabolism after weaning is more intense than that before weaning. L-Gln, an energy source and precursor of endogenous substance (amino acid, nucleic acid, etc.) synthesis in the small intestinal epithelium, is the most abundant non-essential amino acid in mammals [5,8]. In addition, L-Gln is involved in the synthesis of glutathione, which indirectly protects cells from free-radical damage [9]. Therefore, L-Gln is commonly used as a functional antioxidant and energy supplement in the body [10,11]. L-Gln can effectively relieve weaning stress of piglets by increasing feed intake, improving immunity and antioxidant capacity, and increasing the length of small intestinal villus [12,13,14]. This response may be due to the involvement of L-Gln and other compounds in the adaptation and regeneration of intestinal tissues under stress conditions [7,15]. Furthermore, adding L-Gln to piglet diets was found to prevent intestinal atrophy during weaning, because L-Gln can stimulate lymphocyte regeneration and macrophage activity without affecting the normal intestinal structure [16,17]. Some studies also showed that L-Gln added at 1% of milk DM in high-dose milk benefitted the growth of small intestine [18], and addition of L-Gln at 1% of milk replacer DM in high-dose milk replacer enhanced the dry matter intake of calves [19]. Additionally, a recent study reported that supplementing L-Gln with 2% of DMI in 9 L/d milk weaned completely 3 d earlier than calves without L-Gln [20]. However, there are few studies on the effects of L-Gln on weaned calves. Although Hu et al. found that intravenous injection of low or medium concentrations of L-Gln in weaned calves significantly improved their immune capacity using parenteral nutrition technology [21], L-Gln injected directly into the bloodstream can be preferentially utilised by the liver and kidney rather than small intestine. Based on these results, dietary L-Gln supplementation that can effectively reduce negative effects of weaning in calves is unclear. Rumen-protected technology is widely used in ruminant nutrition to protect nutrients through the rumen, allowing the digestion and utilisation of nutrients in the small intestine. Therefore, rumen-protected L-Gln (RPG), as an excellent supply of L-Gln, provides L-Gln directly to the small intestine of calves.

In this study, we hypothesized that L-Gln supplementation in diet could effectively relieve weaning stress in a dose-dependent manner, which is probably due to a dose of L-Gln improving calves’ immunity, antioxidant capacity, and intestinal morphology. Based on the hypotheses, this study was conducted to investigate the effects of L-Gln on the growth performance, immune function, antioxidant capacity, and small intestinal structure of weaned calves by adding RPG to a starter diet before and after weaning. Simultaneously, four different levels of L-Gln were used to determine the optimal supplemental level of L-Gln for weaned calves. 

## 2. Materials and Methods

### 2.1. Animal and Management

A total of 75 male and healthy Holstein calves with similar body weight (BW) and age (54.0 ± 2.68 kg; 42 ± 2.1 d of age) were selected for the experiment. Each calf was housed in an individual pen (3 × 2 × 1.5-m^3^; length × width × height) with sand bedding. The pens were cleaned before 0800 h every day to ensure the health and hygiene of the calves. All calves had the same feeding pattern. Briefly, all calves were drenched with a total of 6 L of colostrum, with 4 L drenched within 1 h and 2 L drenched 6 h after birth. Next, the calves were provided commercial milk replacer (Grober Nutrition, Cambridge, ON, Canada; water content: 4.02%, Crude protein: 22%, ether extract: 17%, lactose: 45%, and L-Gln: 0.468%) 2 times a day. The milk replacer was reconstituted as an emulsion (12.5%, *w*/*v*) using cooled (50–60 °C) boiled water and fed to the calves when the temperature cooled to 39 °C. Meanwhile, in the first week of life, the calves were fed 6 L of emulsion per day; 7 L of emulsion per day in the second week of life; and 8 L of emulsion per day from the third week to the weaning day (56 days of age). Along with the emulsion, a commercial pelleted starter feed (Lian Ying Co., Ltd.; water content: 9.35%, crude protein: 22.41%, crude ash: 6.49%, crude fibre: 8.45%, calcium: 0.97%, phosphorus: 0.63%, L-Gln: 0.116%) and clean water (39 °C) were provided ad libitum from 3 d of age. Subsequently, the calves were weaned at 56 days (d) of age (15 d of experiment) and fed only the starter feed after weaning.

### 2.2. Experimental Design

These 75 calves, according to BW and age, were assigned to one of five groups: supplementing L-Gln at 0 (CON), 1% (1%Gln), 2% (2%Gln), 3% (3%Gln), or 4% (4%Gln) of DMI; each group consisted of 15 calves. The experiment lasted 4 weeks (42 d to 70 d of age of calves); all calves, expect the CON group, were provided L-Gln with a commercial RPG, which was provided by Wansheng Biological Co., Ltd. (Zhongwei, Ningxia, China) and contained 50% L-Gln. In situ ruminal degradability of RPG was 25.3% on average, and in vitro small intestinal degradability of RPG was 87.1% on average in our previous study (unpublished). Three Holstein bull with rumen fistula were selected for the in situ ruminal degradability experiment under 1.3 times the maintenance energy level. The starter feed intake of the calves was measured continuously for 3 d before the experiment, and the added amount of RPG was determined according to the DMI of each calf. In addition, we adjusted the amount of RPG per week according to the change in the levels of DMI in each calf. Except for the Con group, each calf was first fed a mixture of 200 g starter feed and RPG every day, and then remaining starter feed was fed when the RPG was exhausted. 

### 2.3. Feed Intake and Growth Performance Measurement 

To calculate the DMI (milk DMI + starter feed DMI; g/d) of each calf, the remaining and added amounts of starter feed were recorded at 0800 h every day, and body weights of the calves were measured every 7 d thereafter before the morning feeding during the experimental period. The average daily gain (ADG) of the calves was calculated every 7 d. Feed efficiency was calculated as ADG/DMI. The content of L-Gln in the milk replacer and starter feed were detected via ultra-performance liquid chromatography-tandem mass spectrometry (UPLC-MS/MS). The UPLC with an Acquity BEH C_18_ column (50 mm × 2.1 mm, 1.7 µm particle size) (Waters, Milford, MA, USA), and the UPLC system was coupled to a Micromass Xevo TQ-S triple quadrupole mass spectrometer (Waters, Manchester, UK) fitted with an electrospray ionization source in negative mode. The referenced detection method was described by Xiao et al. [22].

### 2.4. Blood Sample Collection and Measurement

Nine healthy calves with similar body weight were selected from each group to collect blood samples from the jugular vein before the morning feeding on 0, 7, 14, 16, 18, 21, and 28 d of the experiment. Each blood sample was collected using 10 mL non-anticoagulant tubes. Next, blood samples in non-anticoagulant tubes were centrifuged at 1500× *g* for 30 min at 4 °C (Tiangen OSE-MP25, Beijing, China) to obtain serum, which was then stored in 1.5 mL centrifuge tubes in liquid nitrogen for further analyses.

The concentrations of serum immunoglobulin (Ig) G, IgA, IgM, D-lactate, cortisol, haptoglobin (HP), and interleukin-8 (IL-8) were determined using respective ELISA kits (Nanjing Jian Cheng Bioengineering Institute, Nanjing, China). Serum glucose (Glu), insulin (INS), and urea nitrogen (SUN) concentrations were determined using an automatic biochemical analyser (Kehua-zy KHB-1280, Shanghai, China). Total antioxidant capacity (T-AOC), superoxide dismutase (SOD), glutathione peroxidase (GSH-PX), malondialdehyde (MDA), and catalase (CAT) activity and L-Gln concentration in the serum were determined using commercial kits (Nanjing Jian Cheng Bioengineering Institute, Nanjing, China). 

### 2.5. Small Intestine Sampling, Processing, and Morphological Analysis

**Figure 1:**
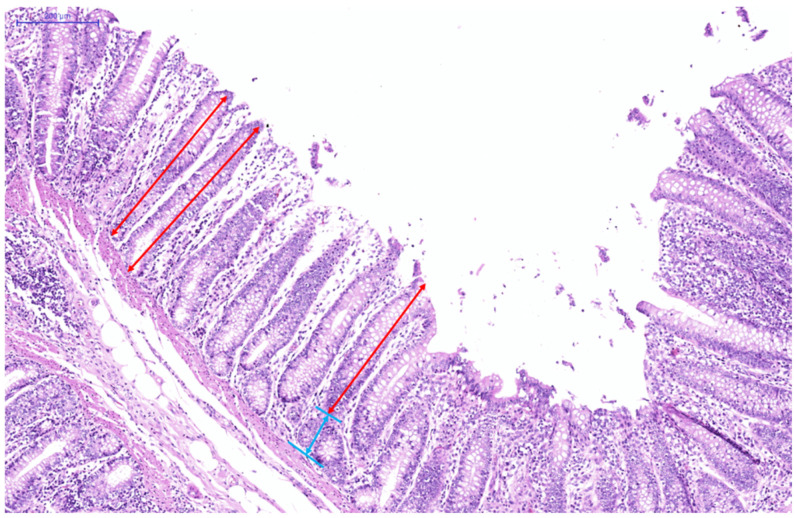
Representative histological pictures of the small intestine, magnified 80×. Red lines represent villus height; blue lines represent crypt depth.

At the end of the experiment, six healthy calves with similar body weight (the calves from whom blood samples had been obtained) were slaughtered from each group. Next, we collected the entire small intestine of the calves, from the pyloric sphincter to the ileocecal valve. The duodenum, jejunum, and ileum samples were collected 6 cm posterior to the pylorus, the middle part of the jejunum, and 6 cm posterior to the jejunum-ileum junction by an animal anatomist. All tissue samples were immediately washed with saline to remove the chyme of the samples then preserved in 10% buffered formalin for analysis of intestinal morphology. After fixation in the buffered formalin, the samples were trimmed to maintain an integrated cross section. Next, the sections were trimmed from each sample by a transversal cut through the villi and dehydrated overnight using graded alcohol (50%, 75%, 85%, 95%, and 100% alcohol) at ambient temperature. The samples were cleared in a mixture of xylene: ethyl alcohol = 1:1) and then impregnated with Histosec paraffin pastilles (Merck Ltd., Auckland, New Zealand). Subsequently, the samples were embedded (KD-BM, Kedi Ltd., Jinghua, China) and cut using a paraffin slicing machine (RM2016, Laika Ltd., Shanghai, China). The 5 μm-thick sections were stained with haematoxylin-eosin until they were no longer faded. Morphometric analysis included the evaluation of villus height and width, crypt depth, villus height/crypt depth (V/C). Measurements were taken from 10 villi per section and 2 sections per calf. The measurements were performed by an investigator blinded to the treatment allocation of the calves using the image processing Pannoramic Viewer software (v 1.15.3, 3DHISTECH Ltd., Budapest, Pest, Hungary); the measurement criteria are described in Figure 1.

### 2.6. Statistical Analyses

Statistical analyses were performed using R software (v. 4.0.3, https://www.r-project.org/, accessed on 25 June 2021). The statistical power for the serum samples in this study was >0.8 with a significance level of 0.05 using G*Power software (v. 3.1.9.6, https://g-power.apponic.com, accessed on 25 June 2021). Before analysis, all data were checked for normality using shapiro.test function of the R software, and the data fitting non-normality was log-transformed. Next, indicators of feed intake, growth performance, serum immunity, and serum antioxidants were analysed via a mixed linear model, as follows:

$$Y_{ijkl}=\mu +T_{i}+D_{j}+TD_{ij}+Calf_{k}+e_{ijkl}$$

where $Y_{ijkl}$ is the dependent variable; μ is the average experimental value; $T_{i}$ is the fixed effect of L-Gln supplementation or not; $D_{j}$ designates the repeated effect; $TD_{ij}$ is the interaction effect of $T_{i}$ and $D_{j}$; $Calf_{k}$ is the random effect of calf; and $e_{ijkl}$ is the error term. Another mixed linear model was used to analyse the intestinal tissue samples; the relevant model is as follows:

$$Y_{ijk}=\mu +T_{i}+Calf_{j}+e_{ijk}$$

where $Y_{ijk}$ is the dependent variable; μ is the average experimental value; $T_{i}$ is the fixed effect of L-Gln supplementation or not; $Calf_{j}$ is the random effect of calf; and $e_{ijk}$ is the error term. The values of all data were reported as least squares means, and polynomial orthogonal contrasts in R software were used to test linear and quadratic responses. Differences of *p* ≤ 0.05 were considered significant, and those of 0.05 < *p* ≤ 0.10 were considered to show a tendency of difference.

## 3. Results

### 3.1. Feed Intake and Growth Performance

With increasing quantity of L-Gln added to the diet, the body weight of the calves in the 4-week experimental period had a quadratically increasing tendency, whereas BW at 1-, 2-, and 3-week experimental periods was not affected (Table 1). Meanwhile, L-Gln supplementation significantly boosted the average daily gain, and increasing the quantity of L-Gln quadratically increased the ADG during the pre-weaning period and total experimental period. We also found that L-Gln supplementation significantly enhanced the DMI, and the DMI had a linear increase during the pre-weaning period and total experimental period. L-Gln intake was significantly affected by L-Gln supplementation, and it showed a linear increase with increasing supplemental levels of L-Gln. In addition, the DMI during the total experimental period showed a quadratically increasing tendency. L-Gln supplementation during the post-weaning experimental period significantly affected the feed efficiency, and the feed efficiency showed a quadratically increasing tendency with increasing supplemental levels of L-Gln. 

### 3.2. Serum L-Gln, Glu, Urea Nitrogen, and Insulin

We found that the concentrations of serum L-Gln and Glu were not different among the five groups during the entire experimental period (Table 2). Interestingly, L-Gln supplementation at 16, 18, and 28 d of experiment and during post-weaning and the whole experiment period significantly enhanced the concentration of SUN. Additionally, we also found that the SUN concentrations showed linear increases with increasing supplemental levels of L-Gln during post-weaning and the whole experiment period; at 16 and 18 d of the experiment, the concentration of SUN showed linear and quadratic increases with increasing supplemental levels of L-Gln. Furthermore, L-Gln supplementation significantly boost INS concentration, and the INS concentration presented linear increasing with increasing L-Gln levels during the pre-weaning period.

### 3.3. Stress-Related Hormone Indicators

To assess the stress status of calves in each group, we measured the concentrations of serum cortisol, HP, IL-8, and D-lactate (Table 3). Serum cortisol concentrations in L-Gln supplementation groups were significantly higher than that in the CON group at 16 and 21 d of experiment, and during the post-weaning period. Meanwhile, they showed a significant increasing tendency compared to the CON group. Furthermore, we also found cortisol concentration quadratically decreased as L-Gln inclusion in the diet increased at 16 and 21 d of experiment and during the post-weaning and the whole experiment period. Although L-Gln supplementation groups had no effect on the concentration of serum HP and IL-8, we found that HP and IL-8 concentrations showed a quadratic decrease with increasing L-Gln levels in the diets at 16 and 21 d of experiment and during the post-weaning period. At 21 d of experiment, L-Gln supplementation groups significantly decreased serum D-lactate concentration, and D-lactate level showed a quadratic decrease with increasing L-Gln levels in the diets. In addition, we also found that D-lactate showed a quadratically decreased tendency with increasing L-Gln levels in the diets during the post-weaning and whole experiment period.

### 3.4. Oxidative Status

Table 4 shows the antioxidant capacity in serum. The T-AOC in L-Gln supplementation groups, during the post-weaning and whole experiment period, were significantly higher than that in the CON group, and it linearly and quadratically increased with L-Gln added to the diets. Furthermore, T-AOC in L-Gln supplementation groups showed a significant difference and tendency compared to that in the CON group, respectively, and it presented a linear increasing and quadratic increasing tendency with increasing L-Gln levels at 14 and 16 d of experiment, respectively. GSH-Px level in L-Gln supplementation groups during the pre-weaning, post-weaning, and whole experiment period were significantly higher than that in the CON group, and it showed a linear and quadratic increase with L-Gln added to the diets. Moreover, we also found that the GSH-Px level in L-Gln supplementation groups at 7, 14, and 16 as well as 18 d of experiment showed a significant increase and increasing tendency, respectively, and it also showed a linear and quadratic increase with L-Gln added to the diets. Although there was no significant difference for SOD between L-Gln supplementation groups and CON, the SOD level showed a quadratic increase with increasing L-Gln level added to the diets at 16 d of experiment. At 16 and 18 d of experiment, the CAT in L-Gln supplementation groups were significantly higher than that in the CON group, and increasing L-Gln level added to diets quadratically increased the CAT level. In contrast, MDA in L-Gln supplementation groups were significantly lower than that in the CON group, and it quadratically decreased with increasing Gln levels at 18 d of the experiment.

### 3.5. Immune Status

Table 5 shows the immune capacity among groups. IgG concentration in L-Gln supplementation groups, during the pre-weaning, post-weaning, and whole experiment period, were significantly higher than that in the CON group, and it showed a linear and quadratic increase with L-Gln supplementation in the diets. We also found that, except for at 0 d of experiment, L-Gln supplementation groups boosted significantly or had a significant trend on IgG concentration at other sampled times. For IgA concentration, it appeared to have a quadratic increasing tendency at 14 d of the experiment. The serum IgM in L-Gln supplementation groups was significantly higher than that in the CON group, and increasing L-Gln level added to the diets quadratically increased the serum IgM concentration at 7 and 14 d of experiment and during the pre-weaning and whole experiment period. Furthermore, the serum IgM concentration showed a linear increase and linear increasing tendency at 7 d of experiment and during the pre-weaning period as well as at 14 d of experiment, respectively.

### 3.6. Small Intestinal Morphology

**Figure 2:**
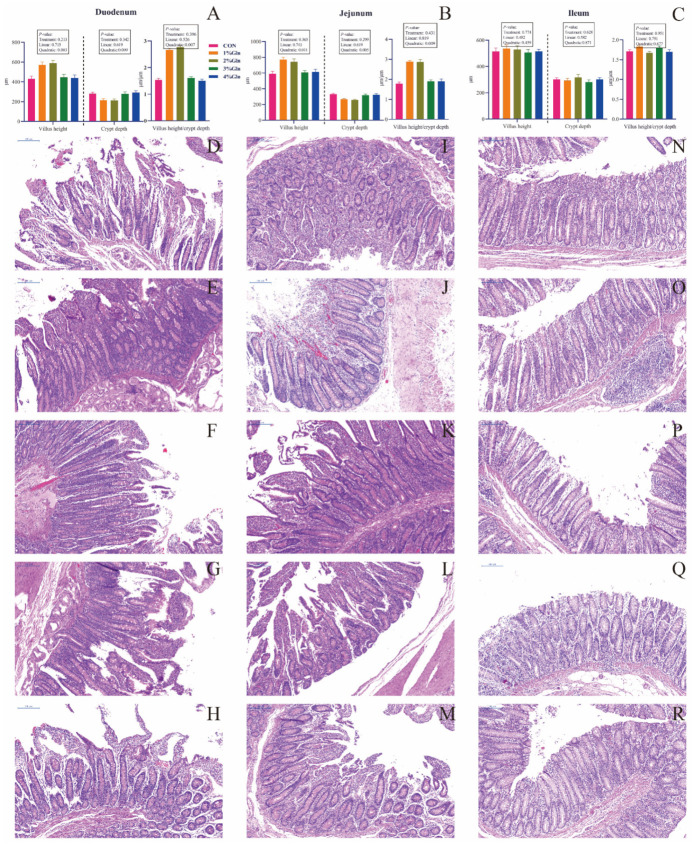
Histomorphology of the small intestine ((**A**): duodenum, (**B**): jejunum, and (**C**): ileum; mean ± SEM) among different groups (N = 6 per group). Light micrographs (80× magnification, blue bar represents 200 µm) of the duodenum (**D**–**H**), Jejunum (**I**–**M**), Ileum (**N**–**R**) of calves in CON (**D**,**I**,**N**) or 1%Gln (**E**,**J**,**O**) or 2%Gln (**F**,**K**,**P**) or 3%Gln (**G**,**L**,**Q**) or 4%Gln (**H**,**M**,**R**).

As shown in Figure 2, we observed that increasing the L-Gln quadratically increased the villus height and V/C of the duodenum and jejunum. In contrast, the crypt depth of the duodenum and jejunum decreased quadratically with increasing L-Gln levels. 

## 4. Discussion

L-Gln, as a functional amino acid, is widely used as a feed additive in pigs [16,17] and chickens [24] to combat stress injury. However, research on glutamine in young ruminants is limited. Considerable development in gastrointestinal physiology occurs during the first few weeks of life, allowing calves to quickly adapt to the liquid–solid dietary transition. During the transition, the calf often experiences weaning stress, which poses great challenges to its health and adult productivity. To our knowledge, this study is the first to examine the effects of rumen-protected L-Gln supplementation in the diet on calves during weaning period. The findings revealed that low L-Gln supplementation (1 and 2% of DMI) in the diet better relieved the negative effects of weaning on calves via enhancing the immune and antioxidant capacity as well as improving intestinal morphology.

Growth performance is a crucial indicator of evaluating weaning stress status; calves with severe weaning stress have lower ADG and feed efficiency [3]. Although the ADG and feed efficiency of weaned calves were lower than pre-weaning in this study, we found that the addition of L-Gln to the diets improved the growth performance of weaned calves, and low L-Gln level supplementation (1 and 2% of DMI) had better effects. Similar to our result, Ma et al. reported that medium L-Gln level (160 g/d) supplementation could promote the compensatory growth of growth-retarded yaks compared to the high level (180 g/d) [25]. Another recent study also found that compared with no supplementation, the ADG of weaned calves at 35 d of age was increased by adding L-Gln with 2% of DMI to diet, and weaned completely 3 d earlier [20]. However, Hu et al. found that different doses of glutamine administered intravenously did not improve the growth performance of calves during the post-weaning period, possibly because intravenous administration of L-Gln first entered the liver and kidney for metabolic utilisation, whereas L-Gln entering the small intestine did not reach the threshold of effective concentration [21]. In addition, we found that L-Gln supplementation also increased the DMI of calves during the pre-weaning and total experiment period, but had no effect on feed efficiency. Da Silva et al. found a similar result when they added L-Gln in milk replacers for calves [19]. These results also indicate that L-Gln has potential as a functional food attractant for calves in the future.

Cortisol and HP are key indicators of reacting stress degree [26,27]. IL-8 is a biomarker of weaning stress [28]. In contrast to DMI and feed efficiency, cortisol, HP, and IL-8 of weaned calves were lower than pre-weaning in this study. Furthermore, they showed a quadratic decrease with increasing L-Gln level added in the diet. In particular, 1 and 6 d after weaning the calves, the lower-level L-Gln (1 and 2% of DMI) groups also had better results, which further confirmed that the lower levels of L-Gln can alleviate the adverse effects of weaning in calves. Interestingly, L-Gln supplementation had no effect on serum L-Gln concentration in this study, which is similar to previous studies about piglets [29,30]. These responses indicate that L-Gln added to the diet will be metabolized by the digestive tract and other organs before entering the bloodstream. Prior to weaning, L-Gln supplementation increased serum insulin concentration and linearly increased with supplemental level, whereas serum glucose concentration was not affected. Wu reported that although L-Gln was essential for the integrity and development of the gut, 90% of it is not utilized efficiently in normal conditions [31]. Combined with the SUN results before weaning, we thought that part of L-Gln, which could not be utilized by the small intestine, might be converted into glucose in blood, and the body maintains glucose homeostasis by raising insulin levels. After weaning, L-Gln supplementation linearly increased SUN concentration. The reason for the results that L-Gln might be conversed to the glutamate and ammonium providing the energy for small intestine by deamination in the small intestine’s mitochondria. The ammonium will eventually contribute to hepatic ureagenesis and therefore to the increase the SUN concentrations. Interestingly, we still found that the lower-level L-Gln (1% and 2% of DMI) had higher SUN concentrations at 1 d after weaning. The result also suggested that the lower-level L-Gln could be utilized efficiently by the small intestine after weaning in this study, which might also be why higher-level L-Gln (3% and 4% of DMI) had no better effect than low-level L-Gln (1% and 2% of DMI) supplementation groups on alleviating the negative effects of sudden weaning.

The negative effect of sudden weaning is usually accompanied by oxidative stress; therefore, the antioxidant capacity of calves can reflect their ability to resist weaning stress. Normally, there is a redox balance between the production of reactive oxygen species in the body’s intracellular environment and the ability of biological antioxidant systems to neutralise these active intermediates [32,33]. Oxidative stress disrupts this balance and causes tissue damage [34]. In turn, the body produces antioxidant enzymes to reduce ROS; for example, GSH-Px is an important antioxidant in the body, and glutamate, the metabolite of glutamine in cells, is one of its main components [35,36]; SOD and CAT can clear free radicals and hydrogen peroxide in cells, respectively [34]; MDA is the oxidation end-product of hydrogen peroxide and can also indirectly reflect the degree of tissue peroxide damage [37]. Therefore, the greater the levels of SOD, CAT, and GSH-Px and the lower the levels of MDA, the stronger the antioxidant capacity of the body. Combined with the results of this study, these results suggest that dietary L-Gln supplementation can improve resistance to oxidative stress in weaned calves, and lower-level groups have better capacity because of higher GSH-Px activity. Similarly, Wu et al. found that glutamate supplementation reduced oxidative stress by inhibiting the mTOR signalling pathway [38]. In fish, glutamate supplementation can increase the activity of GSH-PX and SOD to reduce intestinal oxidative damage [39]. Therefore, we think L-Gln with 1 and 2% of DMI enhance the antioxidant capacity of weaned calves by breaking it down into glutamate, which is consistent with higher SUN concentration at 1 d after weaning. Immunoglobulin, which is present in the serum, is a glycoprotein produced by the proliferation and differentiation of B cells into plasma cells after antigen stimulation. The determination of serum IgG, IgA, and IgM levels can reflect the body’s humoral immunity status. In this study, serum IgM and IgG levels in L-Gln supplementation groups were elevated after weaning. Therefore, we believe that L-Gln supplementation can enhance the immune response of calves, and lower-level L-Gln supplementation can maximise the immune response in this study. IgG, the most abundant immunoglobulin in serum, may be more sensitive to L-Gln stimulation, so it is still significantly elevated after weaning. Hu et al. found that intravenous injection of L-Gln (16 g/d) significantly increased the proportion of CD4^+^ T cells in calves after weaning [21]. CD4^+^ T cells play an important role in the immune system [40]. Thus, intravenous injection of L-Gln (16 g/d) can improve the immunity of calves. Although there is different effect on weaned calves between the parenteral administration and in-feed administration, these results indicate that L-Gln can enhance the immune capacity of weaned calves. Similarly, a recent study found that feeding rumen-protected L-Gln to postpartum cows increased the serum total protein concentration and decreased the somatic cell count of milk [41]. In addition, intravenous injection of L-Gln into postpartum cows also increased the proportion of monocytes and CD8^+^ T cells [42]. In conclusion, low-level L-Gln supplementation can better improve the antioxidant and immune capacities of weaned calves. Interestingly, calves of L-Gln supplementation groups in this study also had better immune and antioxidant capacities before weaning. These results further suggest that L-Gln has potential as a novel antioxidant and immunomodulator for calf production.

Weaning stress causes damage to the small intestine, mainly reflected in the villus becoming shorter and crypt depth becoming higher, and then affects the digestion and absorption of nutrients and body health [43]. In this study, calves fed with 1% and 2% L-Gln had longer villus height, shorter crypt depth, and larger V/C in the duodenum and jejunum. Van Keulen et al. also found that 9 L/d milk allowance with 1% DM of milk L-Gln supplementation significantly increased villus length in the duodenum and jejunum but did not affect crypt depth and V/C [18]. We believe that the difference in the results may be due to the limitations of van Keulen’s study. The addition of L-Gln to 1% DMI of milk may not meet the needs of calves for L-Gln, which is similar to the finding that feeding 1% DMI of milk replacer containing plant proteins L-Gln does not affect the histological morphology of the small intestine [44]. In the present study, we added L-Gln according to the percentage of DMI in the calves. Therefore, compared with the two studies [18,44], we had higher a L-Gln allowance in calves and found that L-Gln added at 2% of total DMI may also be the threshold for small intestine development in calves. Similar to van Keulen et al. [18], no effect of L-Gln on the villus structure of the ileum was found in our study, which may be the reason that L-Gln is mainly absorbed in the duodenum and jejunum. 

Interestingly, high-level L-Gln (3% and 4% of DMI) supplementation groups can relieve the negative effects of weaning; however, there is no better effect than low-level L-Gln (1% and 2% of DMI) supplementation groups. Although similar results have been reported in piglets [29], chickens [24], and yaks [25], the reasons are unclear. Higher-level L-Gln supplementation will release large amounts of ammonium in the gut, and the ammonium can increase metabolic stress of the kidneys and liver, which may damage liver and kidney function; large amounts of ammonium lead to large-scale autophagy of small intestinal cells [45], which may affect nutrient absorption; moreover, it is known that intestinal microbiome plays the crucial role on the utilization of exogenous amino acids, especially epithelial microbiome [31,46,47]. The large amounts of ammonium will alter the pH of the epithelial and lumen environment of intestine, affecting microbial colonization in the small intestine and perhaps causing microbial niche shifts [48]. However, these potential mechanisms need to be verified and explored using molecular biological methods and high-throughput sequencing techniques in the future.

In addition, the rumen-protected L-Gln used in this study was evaluated in situ using Holstein bulls, and the manner was similar to that used in Kong et al. [49]. Holstein bulls have a similar energy allocation to that in Holstein calves, and bulls also consume energy for maintenance and growth rather than for lactation. Calves have a distinct rumen microbiota and morphology from adult cattle [50,51]. The rumen maturity of weaned calves is about 80% of that of adult cattle, and feed degradability in the rumen decreases as DMI rises. Although these factors may influence the true rumen pass rate of the rumen-protected L-Gln in calves, we believe that the deviation does not affect the results of supplementing different levels of L-Gln in this study. Molano et al. found that rumen-protected methionine had a good effect on calves by feeding calves with different methionine sources [52]; Kong et al. also found valid results by feeding calves with rumen-protected lysine [49]. In the research field of rumen-protected products, in vitro and in situ methods are commonly used to evaluate rumen-protected products’ performance, with the in situ method being favoured. In fact, one of the field’s acknowledged limitations is that the degradation rate of rumen-protected products evaluated in adult cattle differs from that of calves; however, calves cannot be used to evaluated for rumen-protected products. As a result, an advanced device that can simulate the rumen motility and environment of calves must be developed in the future.

## 5. Conclusions

The results of this study provide evidence that the addition of L-Gln in the diet improved the negative effects of sudden weaning in calves. Importantly, we found that lower-level L-Gln supplementation (1 and 2% of DMI) in diets had better effect in the growth performance, immune and antioxidant capacity, and morphology of the duodenum and jejunum of weaned calves compared with the higher-level L-Gln supplementation (3 and 4% of DMI) groups. These results provided effective reference for relieving weaning stress of calves. 

## Figures and Tables

**Table 1:** Growth performance and feed intake of calves during different treatments (N = 15 per group).

	Treatment						*p*-Value			
Items	CON	1%Gln	2%Gln	3%Gln	4%Gln	SEM	T	T × D	Linear	Quadratic
BW, kg										
0	57.8	58.0	58.1	56.9	57.7	0.28	0.86		0.64	0.93
1-wk	62.3	63.6	63	61.6	63.3	0.31	0.92		0.99	0.97
2-wk	67.1	68.8	68.6	66.3	68.3	0.32	0.73		0.97	0.64
3-wk	68.6	72.0	72.1	68.3	71.7	0.45	0.21		0.53	0.45
4-wk	72.3	76.6	77.2	72.5	75.3	0.45	0.07		0.62	0.09
ADG, kg/d										
Pre-weaning	0.67	0.77	0.75	0.68	0.76	0.01	0.36	0.29	0.40	0.42
Post-weaning	0.37	0.56	0.61	0.44	0.50	0.02	<0.01	0.12	0.29	0.01
Overall	0.52	0.67	0.68	0.56	0.63	0.01	<0.01	0.61	0.21	0.02
DMI, g/d										
Pre-weaning	1183	1372	1355	1293	1391	14.5	<0.01	0.92	0.01	0.17
Post-weaning	1645	1864	1822	1685	1821	16.3	0.22	0.56	0.17	0.21
Overall	1414	1618	1588	1489	1606	13.3	<0.01	0.42	0.01	0.09
L-Gln intake ^1^, g/d										
Pre-weaning	4.72	18.48	31.86	43.53	60.39	2.35	<0.01	0.77	<0.01	0.17
Post-weaning	0.32	19.00	36.78	50.87	73.19	3.05	<0.01	0.19	<0.01	0.23
Overall	2.52	18.74	34.32	47.20	66.79	2.69	<0.01	0.39	<0.01	0.14
Feed efficiency										
Pre-weaning	0.57	0.56	0.55	0.53	0.55	0.02	0.78	0.95	0.98	0.96
Post-weaning	0.22	0.30	0.34	0.26	0.28	0.03	0.03	0.31	0.37	0.08
Overall	0.37	0.42	0.42	0.38	0.39	0.02	0.26	0.76	0.63	0.21

^1^ L-Gln intake (g/d) = L-Gln intake of milk replacer (milk replacer intake(mL) × 0.125(*w*/*v*, g/mL) × 0.00468) + L-Gln intake of starter feed (feed intake(g/d) × 0.00116× 0.15 (the rumen bypass efficiency of feed protein was 15% in NRC2001 [23])) + L-Gln supplementation (0 or 1% or 2% or 3% or 4% of DMI); BW, body weight; ADG, average daily gain; DMI, dry matter intake; Feed efficiency was calculated as ADG/DMI; T, treatment; D, day.

**Table 2:** Serum Gln, Glu, BUN, and INS indicators of calves among different groups (N = 9 per group).

	Treatment						*p*-Value			
Items	CON	1%Gln	2%Gln	3%Gln	4%Gln	SEM	T	T × D	Linear	Quadratic
L-Gln, mmol/L										
0 d	0.67	0.66	0.68	0.65	0.68	0.02	0.77		0.81	0.57
7 d	0.71	0.76	0.75	0.70	0.74	0.02	0.20		0.37	0.63
14 d	0.72	0.74	0.76	0.73	0.75	0.03	0.93		0.59	0.89
16 d	0.75	0.75	0.77	0.77	0.78	0.02	0.64		0.56	0.79
18 d	0.74	0.78	0.77	0.81	0.79	0.03	0.46		0.31	0.16
21 d	0.80	0.81	0.74	0.80	0.81	0.04	0.64		0.18	0.59
28 d	0.82	0.84	0.81	0.81	0.84	0.04	0.97		0.73	0.63
Pre-weaning	0.70	0.72	0.73	0.69	0.72	0.02	0.39	0.58	0.66	0.29
Post-weaning	0.78	0.80	0.77	0.80	0.81	0.03	0.67	0.69	0.19	0.72
Overall	0.74	0.76	0.75	0.75	0.77	0.01	0.72	0.44	0.97	0.33
Glu, mmol/L										
0 d	6.12	6.16	6.12	6.16	6.14	0.18	1.00		0.98	0.86
7 d	6.18	6.06	5.99	6.04	5.51	0.23	0.29		0.83	0.44
14 d	5.60	5.52	5.68	5.39	5.32	0.12	0.17		0.46	0.45
16 d	4.20	4.36	4.34	4.31	4.32	0.17	0.97		0.61	0.69
18 d	5.23	5.44	4.93	5.09	5.11	0.18	0.35		0.27	0.26
21 d	5.43	5.34	5.20	5.23	5.42	0.14	0.13		0.29	0.43
28 d	5.44	5.33	5.28	5.19	5.22	0.20	0.91		0.57	0.63
Pre-weaning	5.97	5.91	5.93	5.86	5.66	0.15	0.71	0.31	0.51	0.82
Post-weaning	5.08	5.12	4.94	4.96	5.02	0.09	0.33	0.57	0.64	0.46
Overall	5.46	5.46	5.36	5.34	5.29	0.08	0.62	0.39	0.71	0.82
SUN, mmol/L										
0 d	4.54	4.29	4.32	4.30	4.42	0.51	1.00		0.74	0.83
7 d	4.18	4.75	4.41	5.31	5.47	0.79	0.71		0.91	0.31
14 d	2.43	2.69	2.73	2.58	2.82	0.33	0.93		0.74	0.70
16 d	3.31	5.47	5.35	4.38	5.09	0.44	<0.01		0.04	0.05
18 d	8.50	9.67	10.54	12.02	11.51	0.58	<0.01		0.02	0.04
21 d	12.56	12.71	11.99	13.00	12.94	0.79	0.91		0.68	0.45
28 d	8.31	8.85	9.81	10.37	11.18	0.64	0.02		0.31	0.23
Pre-weaning	3.72	3.91	3.82	4.06	4.24	0.53	0.42	0.99	0.26	0.62
Post-weaning	8.17	9.18	9.42	9.94	10.26	0.62	<0.01	0.58	0.02	0.13
Overall	6.26	6.92	7.02	7.42	7.68	0.33	0.04	0.44	0.04	0.14
INS, mIU/L										
0 d	19.49	22.34	19.86	20.58	21.30	1.98	0.84		1.00	0.30
7 d	15.79	18.29	19.32	17.49	18.47	3.45	0.96		0.59	0.86
14 d	14.58	17.51	20.71	15.01	17.69	2.68	0.54		0.28	0.78
16 d	8.73	8.93	10.01	6.50	7.85	1.66	0.65		0.81	0.40
18 d	12.73	18.13	17.08	10.92	13.75	2.11	0.13		0.38	0.87
21 d	14.18	15.56	15.21	13.58	13.98	1.53	0.88		0.73	0.99
28 d	4.76	6.01	5.09	5.54	6.27	0.94	0.77		0.98	0.26
Pre-weaning	16.62	19.38	19.96	17.69	19.15	1.92	0.04	0.72	0.03	0.17
Post-weaning	10.10	12.16	11.85	9.14	10.46	1.73	0.74	0.55	0.48	0.53
Overall	12.89	15.25	15.33	12.80	14.19	0.99	0.15	0.81	0.23	0.19

Glu, glucose; SUN, serum urea nitrogen; INS, insulin; T, treatment; D, day.

**Table 3:** Serum stress indicators of calves among different groups (N = 9 per group).

	Treatment						*p*-Value			
Items	CON	1%Gln	2%Gln	3%Gln	4%Gln	SEM	T	T × D	Linear	Quadratic
D-lactate, μmol/L										
0 d	6.18	6.16	6.30	6.25	6.32	0.05	0.63		0.24	0.86
14 d	6.09	5.98	6.12	6.02	6.13	0.05	0.71		0.78	0.65
16 d	7.52	7.21	7.47	7.82	7.16	0.10	0.83		0.78	0.8
21 d	6.72	6.17	6.03	6.07	6.41	0.07	<0.01		0.12	<0.01
28 d	6.10	6.13	6.00	6.17	6.17	0.06	0.25		0.66	0.61
Pre-weaning	6.14	6.07	6.21	6.14	6.23	0.04	0.97	0.99	0.88	0.79
Post-weaning	6.78	6.50	6.50	6.69	6.58	0.06	0.18	0.58	0.23	0.09
Overall	6.52	6.33	6.38	6.47	6.44	0.03	0.15	0.63	0.14	0.06
Cortisol, μg/dL										
0 d	3.09	3.01	3.22	3.06	2.99	0.11	0.94		0.85	0.72
14 d	3.16	3.19	3.39	3.16	3.18	0.11	0.92		1.00	0.64
16 d	6.72	5.58	5.79	5.85	6.08	0.12	<0.01		0.18	0.01
21 d	5.28	4.29	4.33	4.84	5.13	0.12	<0.01		0.74	<0.01
28 d	3.30	3.20	3.20	3.29	3.33	0.10	0.79		0.85	0.68
Pre-weaning	3.13	3.10	3.31	3.11	3.09	0.10	0.92	0.84	0.77	0.59
Post-weaning	5.10	4.36	4.44	4.66	4.85	0.09	<0.01	0.92	<0.01	<0.01
Overall	4.31	3.85	3.99	4.04	4.14	0.07	0.07	0.36	0.17	0.02
HP, ng/mL										
0 d	0.53	0.52	0.54	0.57	0.51	0.05	0.92		0.77	0.88
14 d	0.54	0.51	0.55	0.52	0.51	0.04	0.88		0.82	0.75
16 d	0.81	0.65	0.67	0.79	0.83	0.05	0.22		0.64	0.03
21 d	0.73	0.62	0.63	0.76	0.74	0.03	0.37		0.83	0.05
28 d	0.56	0.58	0.54	0.61	0.59	0.05	0.83		0.79	0.62
Pre-weaning	0.54	0.52	0.55	0.55	0.51	0.04	0.91	0.92	0.80	0.78
Post-weaning	0.70	0.62	0.61	0.72	0.72	0.04	0.29	0.84	0.63	0.04
Overall	0.63	0.58	0.59	0.65	0.64	0.03	0.34	0.96	0.52	0.17
IL-8, pg/mL										
0 d	50.87	46.07	49.84	47.13	47.92	3.35	0.22		0.50	0.66
14 d	44.14	45.94	47.21	44.01	46.99	2.26	0.35		0.72	0.40
16 d	64.17	49.94	52.88	65.93	64.24	3.13	0.16		0.87	0.03
21 d	58.25	47.46	46.62	55.37	54.29	3.15	0.19		0.27	0.04
28 d	40.89	39.12	41.99	42.75	40.86	1.26	0.47		0.91	0.55
Pre-weaning	47.51	46.01	48.53	45.57	47.46	2.56	0.32	0.58	0.92	0.82
Post-weaning	54.44	45.51	47.16	54.68	53.13	2.02	0.13	0.63	0.25	0.02
Overall	51.66	45.71	47.71	51.04	50.86	1.46	0.29	0.81	0.40	0.22

HP, haptoglobin; IL-8, interleukin-8, T, treatment; D, day.

**Table 4:** Serum antioxidant indicators of calves among different groups (N = 9 per group).

	Treatment						*p*-Value			
Items	CON	1%Gln	2%Gln	3%Gln	4%Gln	SEM	T	T × D	Linear	Quadratic
T-AOC, U/mL										
0 d	23.3	22.2	23.3	26.8	24.6	2.39	0.70		0.77	0.78
7 d	23.2	28.0	28.6	26.1	26.7	2.91	0.32		0.07	0.80
14 d	20.9	27.5	26.1	23.9	26.7	1.79	0.09		0.19	0.09
16 d	18.6	24.2	26.7	26.4	25.8	1.84	0.03		0.01	0.16
18 d	21.4	25.3	24.7	28.7	26.5	2.75	0.48		0.33	0.20
21 d	22.2	28.5	26.3	25.8	26.2	2.13	0.42		0.27	0.15
28 d	22.0	24.5	25.2	25.1	24.6	1.94	0.79		0.29	0.56
Pre-weaning	22.5	25.9	26.0	25.6	26.0	2.04	0.18	0.57	0.11	0.47
Post-weaning	21.1	25.6	25.7	26.5	25.8	1.88	<0.01	0.62	<0.01	<0.01
Overall	21.7	25.7	25.8	26.1	25.9	0.66	<0.01	0.23	<0.01	<0.01
GSH-Px, U/mL										
0 d	60.3	60.9	61.1	63.0	61.8	5.03	1.00		0.86	0.84
7 d	87.1	116.2	112.0	110.2	109.4	6.20	0.04		0.02	0.03
14 d	59.5	87.7	85.7	83.8	85.0	6.50	0.05		0.02	0.04
16 d	47.4	60.3	61.1	52.6	53.4	3.26	0.04		0.02	0.04
18 d	51.4	64.4	67.7	63.0	58.1	8.28	0.07		0.03	0.02
21 d	55.8	66.7	71.6	67.4	64.5	7.48	0.12		0.14	0.08
28 d	60.3	73.1	76.2	69.4	69.4	12.06	0.93		0.43	0.80
Pre-weaning	69.0	88.3	86.3	85.7	85.4	4.64	<0.01	<0.01	<0.01	<0.01
Post-weaning	53.7	66.1	69.2	63.1	61.4	7.52	<0.01	0.82	<0.01	<0.01
Overall	60.3	75.6	76.5	72.8	71.7	2.87	<0.01	0.23	<0.01	0.05
SOD, U/mL										
0 d	73.2	74.6	79.1	75.9	77.0	2.57	0.60		0.22	0.81
7 d	85.1	84.7	83.1	82.0	80.1	1.92	0.77		0.83	0.68
14 d	85.4	86.4	87.0	90.0	86.6	2.56	0.78		0.44	0.63
16 d	89.0	93.8	91.6	93.0	93.1	1.64	0.29		0.34	0.05
18 d	85.2	90.3	91.9	92.4	90.6	3.92	0.73		0.24	0.53
21 d	88.1	91.3	91.6	93.5	90.9	2.05	0.51		0.16	0.34
28 d	80.3	82.1	81.5	83.9	82.3	4.75	0.99		0.79	0.71
Pre-weaning	81.2	81.9	83.1	82.6	81.2	2.04	0.95	0.91	0.84	0.54
Post-weaning	85.7	89.4	89.2	90.7	89.2	3.16	0.76	0.36	0.21	0.52
Overall	83.8	86.2	86.5	87.2	85.8	1.27	0.42	0.66	0.13	0.33
CAT, U/mL										
0 d	28.7	31.9	31.0	29.1	32.3	1.67	0.42		0.80	0.36
7 d	32.1	33.3	34.6	32.3	37.1	2.68	0.63		0.98	0.80
14 d	35.1	35.3	35.3	35.2	38.1	1.70	0.69		0.66	0.63
16 d	35.3	37.4	38.6	38.9	34.6	1.01	0.03		0.17	0.04
18 d	36.8	38.9	38.3	38.4	37.8	1.03	0.04		0.14	0.03
21 d	41.2	42.9	44.4	44.9	44.0	1.37	0.37		0.11	0.52
28 d	43.4	44.3	46.0	49.4	44.9	2.31	0.44		0.21	0.63
Pre-weaning	32.0	33.5	33.6	32.2	35.8	1.93	0.64	0.88	0.48	0.79
Post-weaning	39.2	40.9	41.8	42.9	40.3	0.81	0.11	0.72	0.17	0.12
Overall	36.1	37.7	38.3	38.3	38.4	0.71	0.19	0.92	0.04	0.53
MAD, nmol/mL										
0 d	2.97	2.95	3.03	3.00	2.85	0.13	0.88		0.52	0.59
7 d	3.17	3.27	3.19	3.26	3.20	0.05	0.49		0.56	0.11
14 d	3.18	3.19	3.22	3.20	3.20	0.04	0.97		0.51	0.98
16 d	3.51	3.37	3.29	3.43	3.51	0.09	0.32		0.66	0.06
18 d	3.41	3.28	3.24	3.32	3.27	0.03	0.04		0.57	0.04
21 d	3.15	3.20	3.18	3.14	3.17	0.03	0.67		0.84	0.60
28 d	3.12	3.14	3.16	3.15	3.14	0.02	0.65		0.16	0.75
Pre-weaning	3.11	3.14	3.15	3.15	3.08	0.04	0.66	0.93	0.58	0.65
Post-weaning	3.30	3.25	3.22	3.26	3.27	0.03	0.18	0.94	0.49	0.13
Overall	3.22	3.20	3.19	3.21	3.19	0.02	0.41	0.98	0.13	0.43

T-AOC, total oxidative capacity; GSH–Px, glutathione peroxidase; SOD, superoxide dismutase; CAT, catalase; MDA, malonaldehyde, T, treatment; D, day.

**Table 5:** Serum immune indicators of calves among different groups (N = 9 per group).

	Treatment						*p*-Value			
Items	CON	1%Gln	2%Gln	3%Gln	4%Gln	SEM	T	T × D	Linear	Quadratic
IgG, g/L										
0 d	10.78	11.71	10.76	11.26	12.11	1.32	0.93		0.84	0.46
7 d	4.14	5.72	5.05	4.38	4.78	0.41	0.09		0.30	0.17
14 d	4.65	6.95	6.31	6.24	6.74	0.49	0.02		0.09	0.01
16 d	2.62	4.47	4.02	3.85	3.75	0.40	0.04		0.03	0.04
18 d	1.80	3.09	2.85	2.73	2.80	0.21	<0.01		0.01	<0.01
21 d	2.29	4.08	3.92	3.67	3.74	0.31	<0.01		<0.01	0.01
28 d	5.11	7.84	7.85	7.29	7.30	0.74	0.09		0.03	0.13
Pre-weaning	6.52	8.13	7.37	7.29	7.88	0.55	<0.01	0.58	<0.01	<0.01
Post-weaning	2.96	4.87	4.66	4.39	4.40	0.33	<0.01	0.31	<0.01	<0.01
Overall	4.48	6.27	5.82	5.63	5.89	0.30	<0.01	0.52	0.02	<0.01
IgA, g/L										
0 d	0.81	0.78	0.81	0.80	0.77	0.01	0.29		0.55	0.08
7 d	0.88	0.80	0.90	0.87	0.87	0.03	0.30		0.83	0.07
14 d	0.68	0.74	0.74	0.75	0.73	0.02	0.26		0.10	0.13
16 d	0.71	0.79	0.74	0.74	0.73	0.03	0.27		0.57	0.12
18 d	0.82	0.83	0.80	0.83	0.87	0.05	0.86		0.55	0.45
21 d	0.70	0.70	0.69	0.71	0.69	0.02	0.86		0.94	0.84
28 d	0.74	0.79	0.80	0.80	0.80	0.03	0.53		0.20	0.40
Pre-weaning	0.79	0.77	0.82	0.81	0.79	0.02	0.29	0.91	0.42	0.55
Post-weaning	0.74	0.78	0.76	0.77	0.77	0.03	0.79	0.88	0.34	0.42
Overall	0.76	0.78	0.78	0.79	0.78	0.01	0.74	0.91	0.31	0.49
IgM, g/L										
0 d	2.39	2.30	2.31	2.29	2.36	0.08	0.85		0.33	0.62
7 d	2.36	2.65	2.88	2.75	2.80	0.11	0.03		0.01	0.40
14 d	2.35	2.55	2.53	2.71	2.58	0.08	0.05		0.06	0.03
16 d	2.45	2.69	2.50	2.55	2.56	0.08	0.20		0.69	0.04
18 d	2.64	2.79	2.73	2.77	3.02	0.14	0.43		0.85	0.25
21 d	2.57	2.57	2.51	2.60	2.57	0.07	0.94		0.69	0.62
28 d	2.60	2.68	2.67	2.69	2.67	0.09	0.95		0.58	0.58
Pre-weaning	2.37	2.50	2.57	2.58	2.58	0.09	<0.01	0.56	<0.01	<0.01
Post-weaning	2.57	2.68	2.60	2.65	2.71	0.06	0.37	0.63	0.36	0.51
Overall	2.48	2.60	2.59	2.62	2.65	0.04	0.02	0.47	0.11	0.01

T, treatment; D, day.

## Data Availability

Data is contained within the article.
